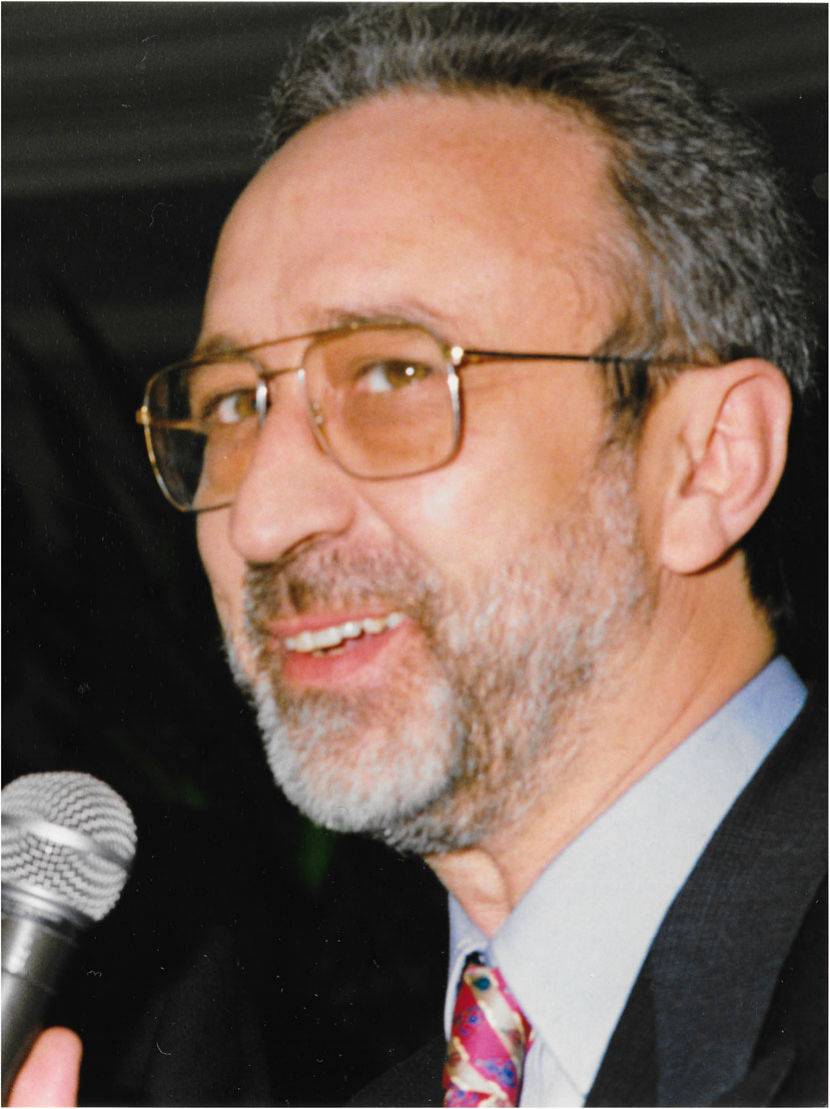# Gian Franco Bottazzo, 1946–2017

**DOI:** 10.1007/s00125-017-4486-x

**Published:** 2017-10-30

**Authors:** Ezio Bonifacio, Emanuele Bosi, R. David Leslie

**Affiliations:** 1 Carl Gustav Carus Faculty of Medicine, DFG-Center for Regenerative Therapies Dresden, Technische Universität Dresden, Fetscherstrasse 105, 01307 Dresden, Germany; 2 Diabetes Research Institute, San Raffaele Vita-Salute University, Milan, Italy; 3 Department of Immunobiology, Blizard Institute, Queen Mary University of London, London, UK

Professor Gian Franco Bottazzo (‘Franco’) left this stage on the evening of September 16th 2017 and his departure brought the curtain down on a small group of individuals who transformed our understanding of the nature of diabetes. Franco died suddenly from complications of endocarditis in his beloved Venice, at the age of 71. Born in Venice, he obtained his MD degree in 1971 at the nearby University of Padua. Shortly after, fate drew him to London to join one of the brightest and most generous of stars in the firmament, the scientist Deborah Doniach. He began a research fellowship with Professor Doniach at Middlesex Hospital and remained in London for over 20 years, becoming a senior lecturer at Middlesex Hospital, then Professor of Immunology, in 1991, at the Royal London Hospital Medical School. He returned to Italy in 1998, taking the role of Scientific Director of the paediatric hospital Bambino Gesù, in Rome, until 2010.

It was early in his fellowship, in 1974, that Franco discovered islet cell antibodies (ICA), leading to a landmark publication in the field of diabetes [1]. This was at a time when a distinction between different forms of diabetes was unclear and the notion that there was an autoimmune form of diabetes was still only a hypothesis. Franco was working with a precious set of samples from patients with multiple endocrine organ-specific autoimmune diseases, some of whom had diabetes mellitus. These samples were what the Doniach laboratory called ‘polyendocrine sera’. The sera fuelled the careers of many fellows as they became a rich source for new antibodies against various cells from a range of organs, such as the pituitary gland, the hypothalamus, the adrenal gland, the intestine and, of course, the pancreas. Franco took on the task of looking for autoantibodies against the pancreas. He obtained some frozen human pancreas tissue and did what he always did best, immunofluorescence. The islets lit up, as Franco would later say, ‘like the Venetian islands at night’, remarking ‘who else but a Venetian could discover ICA?’ Hypothesis became reality and Franco had won the race to change our understanding of type 1 diabetes aetiology. At the age of 28, he became an international figure who brought excitement, innovation (together with the inevitable controversy) and joy to science.

ICA opened up a new area of diabetes research and another seminal discovery followed. Together with Andrew Cudworth, who founded the Barts-Windsor Family Study in order to understand the genetics of diabetes, Franco measured ICA in samples from relatives of individuals with type 1 diabetes. In 1981, they reported that ICA was detectable years before the clinical onset of disease [2], a finding that eventually led to the notion that type 1 diabetes was a chronic disease, which was famously put onto paper by George Eisenbarth [3]. The major targets of these autoantibodies, insulin, GAD_65_, islet antigen 2 (IA-2) and zinc transporter 8 (ZnT8), were slowly discovered in the 80s, 90s and in the new millennium. Eventually, ICA and their target antibodies became part of the classification of type 1A diabetes and were used to distinguish an autoimmune form of diabetes in adults. Moreover, ICA were shown to have value in identifying individuals who would later develop type 1 diabetes [4], providing new staging for presymptomatic type 1 diabetes and enabling clinical trials aimed at delaying the clinical onset of diabetes. Part of this success can be attributed to the standardisation programme set up in 1984; a plethora of methods to measure ICA had appeared and something needed to be done before the field discredited itself by controversy. Åke Lernmark called for an ICA standardisation workshop and Franco established this as part of the Immunology of Diabetes Workshops [5]. This has enabled us to quickly identify diabetes-relevant (and -irrelevant) autoantibodies ever since.

In 1983, Franco, together with fellows Ricardo Pujol-Borrell and Toshi Hanafusa, and with Marc Feldmann, boldly put forward the notion that insults, such as viruses, could induce cells to aberrantly express HLA molecules and that this could lead to organ-specific autoimmunity via de novo and increased self-presentation to T cells [6]. Franco’s exuberance regarding aberrant HLA expression was further aroused after a young physician, Valerie Kitchen, from Leicester (UK) called him to say that a child had tragically died at the onset of diabetes, asking whether he would be interested in looking at the pancreas that had been taken at autopsy. Franco personally drove to collect the pancreas, and he and his team, Betty Dean and Jessica McNally, got to work. They found a few insulin-containing islets with insulitis, and, for the first time, they saw HLA class I hyperexpression on the islet cells, along with what appeared to be beta cells that were also positive for HLA class II [7]. While some holes in the hypothesis surrounding HLA class II expression eventually appeared, the heightened presentation of antigen by the beta cell to T cells remains relevant today and represents a key argument for the continued pursuit of the viral aetiology of type 1 diabetes.

Franco’s findings became legendary through his lecture, ‘Death of a beta cell: homicide or suicide?’ [8], a title that has been borrowed on other occasions [9]. First presented when he received the R. D. Lawrence Lecture Award from the British Diabetic Association in 1985, the homicide or suicide lecture was Franco’s entrance to the ‘stage’. It merged Franco’s fascination for type 1 diabetes with his showman character. Franco, dressed as a barrister in a law court setting, with double slide projection and an animated computer show, led the audience through a court case in which the prosecution tried to convince us that the immune system was guilty of killing the beta cell, while the council for defence presented the case that the beta cell had done everything to present itself to the immune system as something to eliminate. There was also a jury, which pronounced an open verdict (as it probably still would today), an outcome unique to the British legal system as an extension of that British invention, the honourable draw. It was brilliant and the likes of it had not been seen before, or since. Standing ovations led to multiple ‘homicide or suicide’ appearances throughout the world, perhaps the most notable being Franco’s lecture when awarded the Díaz Cristóbal Prize by the International Diabetes Federation in 1985. Franco also received the Oskar Minkowski Prize from the EASD in 1982, the King Faisal International Prize in 1986, and the Banting Medal for Scientific Achievement from the American Diabetes Association in 1992. The Banting lecture provided Franco with the opportunity to stage his ‘walk with Socrates’ production, complete with a supporting cast, at the American Diabetes Association meeting in San Antonio (TX, USA) [10]; Franco rarely did things that were ordinary.

Franco stood at the forefront of type 1 diabetes research for over two decades. His laboratory was a magnet for all to come and try their fortune as a graduate student or post-doctoral fellow. Scientists came from many countries, with multiple languages, races and creeds; an impressive lesson on the value of diversity for developing new ideas and, also, oneself. Franco blended these individuals, from different cultures and with different faiths, without discrimination. His own family, his wife Lamya, from Kuwait, and their daughter Dana, exemplified this passion for life in whichever corner of the world it could be found.

It was a joy to be with him, be it in a microscope room looking at a section of pancreas, or discussing hypotheses of type 1 diabetes aetiology, whether it be the role of cow’s milk or a virus, or his latest unifying hypothesis – conversations we had the pleasure of having with him, even in his last months. Irrepressible, innovative, with a fund of ideas and a treasure trove of historical knowledge, we learned from him how to question everything based on evidence. This was his art: to develop new ideas and then challenge them until only a few of these ideas remained viable. He brought this challenging attitude to every meeting he attended, creating lively discussions, debates and rivalry.

Franco was a friend to many people in the diabetes community, including patients, associations and foundations, and, to the end, he remained engaged with his fellows and friends and the vexing question: why we are still unable to prevent type 1 diabetes? His act may be over but no one was more keenly aware that the show would continue to its inevitable and satisfactory conclusion. He will be missed by his wife Lamya, his daughter Dana and by all those who were fortunate enough to meet him.